# Organ‐Specific Responses to Nivolumab Plus Ipilimumab in Advanced Hepatocellular Carcinoma: A Multicenter, Retrospective Study

**DOI:** 10.1002/cam4.70997

**Published:** 2025-06-09

**Authors:** Jung Sun Kim, Youngun Kim, Beodeul Kang, Ilhwan Kim, Hyeyeong Kim, Won Suk Lee, Jung Yong Hong, Ho Yeong Lim, Han Sang Kim, Chang Gon Kim, Sanghoon Jung, Chansik An, Chan Kim, Hong Jae Chon

**Affiliations:** ^1^ Medical Oncology, Department of Internal Medicine CHA Bundang Medical Center, CHA University School of Medicine Seongnam Korea; ^2^ CHA University School of Medicine Seongnam Korea; ^3^ Division of Oncology, Department of Internal Medicine Haeundae Paik Hospital, Inje University College of Medicine Busan Korea; ^4^ Department of Internal Medicine Ulsan University Hospital, University of Ulsan College of Medicine Ulsan Korea; ^5^ Division of Hematology‐Oncology, Department of Medicine Samsung Medical Center, Sungkyunkwan University School of Medicine Seoul Korea; ^6^ Yonsei Cancer Center, Division of Medical Oncology, Department of Internal Medicine Yonsei University College of Medicine Seoul Korea; ^7^ Department of Radiology CHA Bundang Medical Center, CHA University School of Medicine Seongnam Korea

**Keywords:** advanced HCC, immune checkpoint inhibitor, Nivolumab monotherapy, Nivolumab plus ipilimumab, organ‐specific objective response rate, prior ICI treatment

## Abstract

**Background:**

Immune checkpoint inhibitor (ICI) monotherapy elicits limited intrahepatic responses in patients with advanced hepatocellular carcinoma (HCC). Here, we investigate the organ‐specific objective response rate (OSORR) of nivolumab plus ipilimumab (Nivo/Ipi) combination treatment, considering prior ICI exposure, compared with nivolumab (Nivo) monotherapy.

**Methods:**

We analyzed 204 lesions from Nivo/Ipi‐treated and 305 lesions from Nivo‐treated patients with advanced HCC at five referral cancer centers in Korea. Organ‐specific response criteria were adopted from Response Evaluation Criteria in Solid Tumors 1.1, according to the indicated sites: the liver, lung, lymph nodes (LNs), and other metastatic sites.

**Results:**

Nivo/Ipi combination therapy showed OSORRs of 18.1% in the liver, 17.7% in the lungs, 30.0% in LNs, and 12.5% in other metastatic sites. Patients without prior ICI exposure had OSORRs of 29.0% in the liver, 31.3% in the lungs, 33.3% in LNs, and 23.1% in other metastatic sites (72 individual lesions). Conversely, patients with prior ICI exposure had OSORRs of 11.5% in the liver, 11.4% in the lung, 27.8% in LNs, and 7.4% in other metastatic sites (132 individual lesions). Furthermore, patients who achieved a response in the liver or the lung had longer progression‐free and overall survival, compared with those without responses. Nivo monotherapy yielded OSORRs of 13.5%, 25.3%, 39.3%, and 18.4% in the liver, lungs, LNs, and other metastatic sites, respectively.

**Conclusion:**

Nivo/Ipi combination therapy induced superior intrahepatic responses compared to Nivo monotherapy in patients with advanced HCC without prior ICI exposure, highlighting its potential to overcome liver‐specific immune tolerance.

## Graphical abstract

Organ‐Specific Response to Nivolumab Plus Ipilimumab in Advanced Heaptocellular Carcinoma.

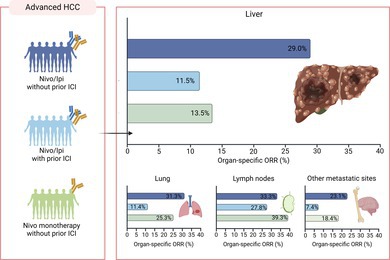

**Abbreviations**
CTLA: cytotoxic T‐lymphocyte‐associated protein
DCR: disease control rate
HBV: hepatitis B virus
HCC: hepatocellular carcinoma
ICI: Immune checkpoint inhibitor
Nivo: nivolumab
Nivo/Ipi: Nivolumab + Ipilimumab
ORR: objective response rate
OS: overall survival
OSORR: organ‐specific objective response rate
PD: programmed cell death
PFS: progression‐free survival
PVTT: portal vein tumor thrombosis
Tregs: regulatory T‐cells

## 1 Introduction

Immune checkpoint inhibitors (ICIs) have reshaped the treatment of various solid malignancies, resulting in significant and durable clinical responses [1, 2, 3, 4]. Despite these advancements, the efficacy of ICI therapy is influenced by the heterogeneity and spatial dynamics of antitumor immune responses within and between tumors [5, 6].

The liver is an inherently immunologically tolerant organ that manages hypersensitivity to dietary and microbial antigens entering through the portal vein [7]. This immune tolerance is crucial for maintaining homeostasis but can also contribute to reduced antitumor immunity [8, 9]. Emerging evidence suggests that intrahepatic tumor lesions often have reduced response rates and poorer survival outcomes to ICIs, supporting potential liver‐specific immune tolerance. Recent preclinical studies have demonstrated that the presence of tumor antigens in the liver suppresses systemic antitumor immune responses. This antigen‐specific immune suppression is mediated by regulatory T‐cells (Tregs) and intratumoral CD11b + monocytes and, thus, cannot be reversed by programmed cell death (PD)‐1 blockade alone; instead, it can be overcome by Treg depletion through anti‐cytotoxic T‐lymphocyte‐associated protein (CTLA)‐4 blockade [10, 11].

Given that hepatocellular carcinoma (HCC) originates in the liver, the immune‐tolerant microenvironment of the liver may significantly undermine immunotherapeutic efficacy within the hepatic tumor microenvironment [12, 13]. Phase III clinical trials of anti‐PD‐1 monotherapy in advanced HCC reported low objective response rates (~15%), with particularly worse responses in intrahepatic lesions, suggesting the potential involvement of liver‐specific immune tolerance [14, 15].

Recent phase III trials, such as CheckMate 9DW and HIMALAYA trials, have demonstrated that combination immunotherapy with anti‐PD‐1/PD‐L1 and anti‐CTLA‐4 antibodies can improve overall survival (OS) in advanced HCC [16]. However, to the best of our knowledge, no study has specifically addressed the organ‐specific response of intrahepatic lesions to anti‐PD‐1/PD‐L1 and anti‐CTLA‐4 combination immunotherapy. In this study, we aimed to determine whether the nivolumab plus ipilimumab (Nivo/Ipi) combination is more effective than nivolumab (Nivo) monotherapy in controlling intrahepatic lesions, whether its efficacy is influenced by prior ICI exposure, and whether this organ‐specific response affects OS in advanced HCC.

## 2 Methods

### 2.1 Patients

**FIGURE 1:**
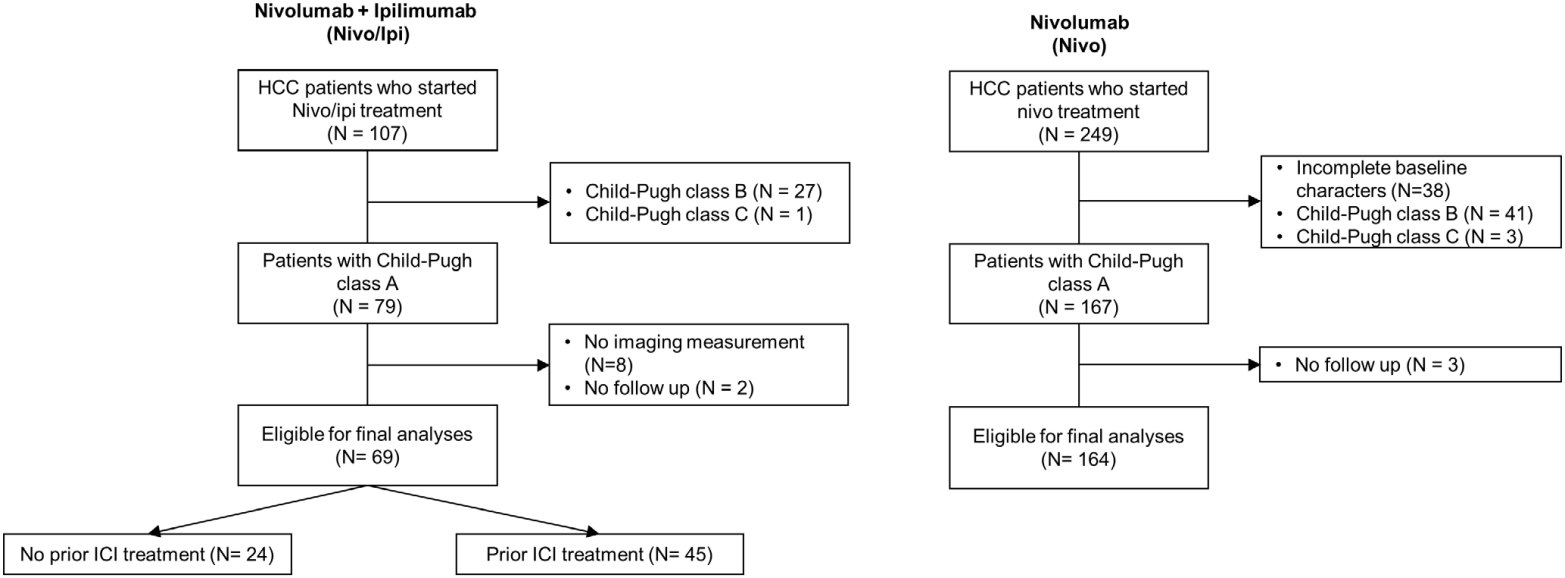
CONSORT diagram.

This retrospective study analyzed patients with advanced HCC treated with either Nivo/Ipi (*n* = 107), initiated between March 2020 and September 2023, or Nivo monotherapy (*n* = 249), initiated between June 2012 and March 2018, at five referral cancer centers in Korea (CHA Bundang Medical Center, Haeundae Paik Hospital, Samsung Medical Center, Ulsan University Hospital, and Yonsei Medical Center) (Figure 1). Patients with no target lesion according to Response Evaluation Criteria in Solid Tumors (RECIST) version 1.1, those classified as Child‐Pugh Class B or C, and those lacking baseline clinical or follow‐up data were excluded. A total of 69 patients in the Nivo/Ipi group and 164 patients in the Nivo monotherapy group were included in the final analysis. The median follow‐up periods were 7.1 months for the Nivo/Ipi group and 6.2 months for the Nivo monotherapy group.

This study was approved by the Institutional Review Boards (CHA Bundang Medical Center, 2019‐10‐009, 2024‐07‐063; Yonsei Cancer Center, 4‐2019‐0882; Samsung Medical Center, 2019‐10‐063; Haeundae Paik Hospital, 2019‐10‐025‐001; and Ulsan University Hospital, 2019‐11‐031‐002) of each participating institution and was conducted in accordance with the ethical guidelines of the Declaration of Helsinki.

### 2.2 Treatment and Assessment

Patients in the Nivo/Ipi group received intravenous nivolumab (1 mg/kg) plus ipilimumab (3 mg/kg) every 3 weeks for up to four doses, followed by nivolumab 240 mg every 2 weeks, in accordance with the CheckMate‐040 trial [17]. In the Nivo monotherapy group, patients received intravenous nivolumab 200 mg every 2 weeks, in accordance with the CheckMate‐459 trial [15]. The treatment was continued until unacceptable toxicity, disease progression, or death occurred. Dose reductions or interruptions were made at the discretion of the attending physician, based on the patient's medical condition. Tumor responses were evaluated every 6–9 weeks using computed tomography according to RECIST version 1.1.

### 2.3 Organ‐Specific Objective Response Rate (OSORR)

OSORRs were evaluated based on RECIST version 1.1 by applying the measurable lesion assessment criteria to each organ individually at each computed tomography (CT) imaging time point. Measurable lesions include those in the liver, lungs, lymph nodes (LNs), and other metastatic sites, such as the brain or bones. According to the RECIST guidelines, lesions were considered measurable if they had a long axis of at least 10 mm, while measurable LNs were defined as those with a short axis of at least 15 mm. Target lesions were selected with a maximum of two lesions per organ and up to five lesions in total per patient, as specified in the RECIST guidelines. The presence of new lesions was not taken into account in the assessment of OSORR. All the images were independently and anonymously reviewed by two board‐certified radiologists (S.J. and C.A.).

### 2.4 Statistical Analysis

Statistical analyses were conducted using SPSS software version 22 (IBM Corp., Armonk, NY, USA) or R software version 4.4.1 (R Foundation for Statistical Computing, Vienna, Austria). The Chi‐square test or Fisher's exact test was utilized to compare categorical variables, while the Student's *t*‐test was utilized for continuous variables. The Kaplan–Meier method and log‐rank test were used to compare survival outcomes between the groups. Statistical significance was set at *p* < 0.05. The graphs were created utilizing Prism 8.0 software (GraphPad Software Inc., San Diego, CA, USA).

## 3 Results

### 3.1 Patient Characteristics

In this study, we analyzed 204 individual lesions in 69 patients who were treated with Nivo/Ipi, and 305 individual lesions in 164 patients who were treated with Nivo monotherapy (Figure 1 and Table 1).

**TABLE 1:** Demographic characteristics of the patients.

*N* (%)	Nivolumab + Ipilimumab			Nivolumab (*N* = 164)	*p*
	Total (*N* = 69)	Prior ICI (*N* = 45)	No Prior ICI (*N* = 24)		
Age (median, IQR)	60 (53–65)	60 (54–65)	59 (52–64)	59 (52–68)	0.707
Gender					0.694
Male	57 (82.6)	36 (80.0)	21 (87.5)	138 (84.1)	
Female	12 (17.4)	9 (20.0)	3 (12.5)	26 (15.9)	
Etiology of HCC					0.204
Hepatitis B	56 (81.2)	39 (86.7)	17 (70.9)	122 (74.4)	
Hepatitis C	3 (4.3)	0 (0.0)	3 (12.5)	12 (7.3)	
Alcohol	6 (8.7)	4 (8.9)	2 (8.3)	9 (5.5)	
MASLD	4 (5.8)	2 (4.4)	2 (8.3)	21 (12.8)	
ECOG performance status					0.246
0	6 (8.7)	2 (4.4)	4 (16.7)	18 (11.0)	
1	63 (91.3)	43 (95.6)	20 (83.3)	146 (89.0)	
Child‐Pugh Score					< 0.001
A5	30 (43.5)	23 (51.1)	7 (29.2)	112 (68.3)	
A6	39 (56.5)	22 (48.9)	17 (70.8)	52 (31.7)	
BCLC stage					0.795
B	4 (5.8)	2 (4.4)	2 (8.3)	11 (6.7)	
C	65 (94.2)	43 (95.6)	22 (91.7)	153 (93.3)	
Extrahepatic metastasis					0.800
No	9 (13.0)	5 (11.1)	4 (16.7)	23 (14.0)	
Yes	60 (87.0)	40 (88.9)	20 (83.3)	141 (86.0)	
Portal vein tumor thrombosis					0.053
No	54 (78.3)	38 (84.4)	16 (66.7)	108 (65.9)	
Yes	15 (21.7)	7 (15.6)	8 (33.3)	56 (34.1)	
Line of therapy					< 0.001
≤ 3rd line	36 (52.2)	16 (35.6)	20 (83.3)	153 (93.3)	
3rd line	33 (47.8)	29 (64.4)	4 (16.7)	11 (6.7)	

Abbreviations: BCLC, Barcelona clinical liver cancer stage; ECOG, Eastern Cooperative Oncology Group; HCC, hepatocellular carcinoma; IQR, interquartile range; MASLD, metabolic dysfunction‐associated steatotic liver disease; N, numbers.

The median age was 60 and 59 years in the Nivo/Ipi and Nivo groups, respectively. In both groups, most patients were male (82.6% in the Nivo/Ipi group vs. 84.1% in the Nivo group), and the most common etiology of HCC was hepatitis B virus (HBV) infection (81.2% in the Nivo/Ipi group vs. 74.4% in the Nivo group). Most patients had an Eastern Cooperative Oncology Group performance status of 1 (91.3% in the Nivo/Ipi group vs. 89.1% in the Nivo group). Child‐Pugh Class A5 was observed in 43.5% of the patients in the Nivo/Ipi group, and 68.3% in the Nivo group. Most patients in both groups were classified as Barcelona Clinic Liver Cancer stage C (94.2% in the Nivo/Ipi group vs. 93.3% in the Nivo group). Extrahepatic metastasis was present in 87.0% of patients in the Nivo/Ipi group and 86.0% in the Nivo group. Portal vein tumor thrombosis (PVTT) was observed in 21.7% and 34.1% of patients in the Nivo/Ipi and Nivo groups, respectively. Notably, 65.2% of the patients in the Nivo/Ipi group had a history of prior ICI treatments, compared to only 3.7% in the Nivo group. Of those in the Nivo/Ipi group with prior ICI 64.4% had previously received atezolizumab plus bevacizumab and 24.4% had received Nivo.

When comparing patients in the Nivo/Ipi groups based on prior ICI exposure, the clinical characteristics were generally similar except for the Child‐Pugh score. In the Nivo/Ipi group with prior ICI exposure, 51.1% were classified as A5 and 48.9% as A6, whereas in the group without prior ICI exposure, 29.2% and 70.8% were classified as A5 and A6, respectively. Among patients with prior ICI exposure, 15.6% had PVTT compared to 33.3% in those without prior ICI exposure.

### 3.2 Organ‐Specific Responses in the Nivo/Ipi Treatment Group and the Nivo Monotherapy Group

We analyzed the overall treatment response of patients in the Nivo/Ipi and Nivo groups during the follow‐up period (Table 2). The Nivo/Ipi group had an objective response rate (ORR) of 29.0% and a disease control rate (DCR) of 40.6%. Patients with prior ICI exposure had an ORR of 20.0% and a DCR of 33.3%, whereas those without prior ICI exposure had an ORR of 45.8% and a DCR of 54.1%. In the Nivo group, the ORR was 21.3% and the DCR was 54.3%.

**TABLE 2:** Patient‐based best overall response.

*N* (%)	Nivolumab + Ipilimumab			Nivolumab (*N* = 164)	*p* *
	Total (*N* = 69)	Prior ICI (*N* = 45)	No Prior ICI (*N* = 24)		
Best response					
CR	4 (5.8)	2 (4.4)	2 (8.3)	3 (1.8)	
PR	16 (23.2)	7 (15.6)	9 (37.5)	32 (19.5)	
SD	8 (11.6)	6 (13.3)	2 (8.3)	54 (32.9)	
PD	41 (59.4)	30 (66.7)	11 (45.9)	75 (45.8)	
ORR	29.0	20.0	45.8	21.34	0.019
DCR	40.6	33.3	54.1	54.27	1.000

Abbreviations: CR, complete response; DCR, disease control rate; ICI, immune checkpoint inhibitor; Nivo/Ipi, Nivolumab + Ipilimumab; Nivo, Nivolumab; ORR, objective response rate; PD, progressive disease; PR, partial response; SD, stable disease.

* *p*‐values were calculated using Pearson's chi‐square test.

To assess how these responses apply to each organ, we compared the OSORRs among the organs (Figures S1 and 2, Table 3). Nivo/Ipi combination treatment induced OSORRs: 18.1% (15/83) in the liver, 17.7% (9/51) in the lungs, 30.0% (9/30) in LNs, and 12.5% (5/40) at other metastatic sites (204 individual lesions). In the Nivo/Ipi group, patients with prior ICI exposure exhibited OSORRs: 11.5% (6/52) in the liver, 11.4% (4/35) in the lungs, 27.8% (5/18) in LNs, and 7.4% (2/27) in other metastatic sites (132 individual lesions). Patients without prior ICI exposure had higher OSORRs: 29.0% (9/31) in the liver, 31.3% (5/16) in the lungs, 33.3% (4/12) in LNs, and 23.1% (3/13) in other metastatic sites (72 individual lesions). In the Nivo group, patients had OSORRs: 13.5% (21/156) in the liver, 25.3% (21/83) in the lungs, 39.3% (11/28) in LNs, and 18.4% (7/38) at other metastatic sites (305 individual lesions). This suggests that each organ exhibited a superior response in patients without prior ICI exposure compared to those with prior ICI exposure and those receiving Nivo monotherapy.

**FIGURE 2:**
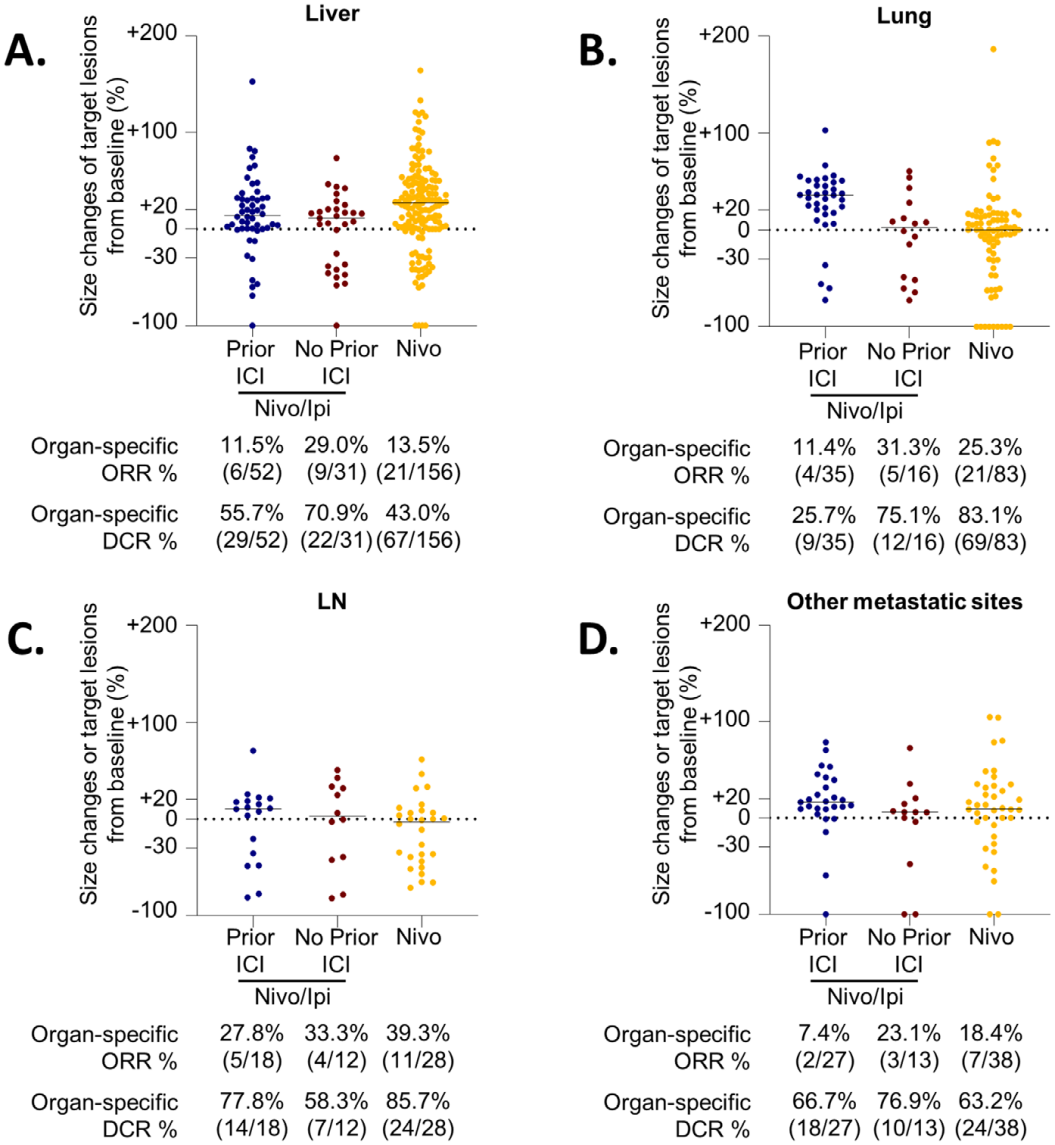
Organ‐specific response based on the treatment strategy. (A) Liver‐specific response (B) Lung‐specific response (C) Lymph node‐specific response (D) Other metastatic lesion (bone and brain)‐specific response. DCR, Disease Control Rate; ICI, Immune checkpoint inhibitor; Nivo/Ipi, Nivolumab plus Ipilimumab; Nivo, Nivolumab; ORR, Objective Response Rate.

**TABLE 3:** Best organ‐specific response.

*N* (%)	Nivolumab + Ipilimumab			Nivolumab *N* = 156	*p* *
	Total	Prior ICI	No prior ICI		
Liver	*N* = 83	*N* = 52	*N* = 31	*N* = 156	
CR	2 (2.4)	1 (1.9)	1 (3.2)	5 (3.2)	
PR	13 (15.7)	5 (9.7)	8 (25.8)	16 (10.3)	
SD	36 (43.4)	23 (44.2)	13 (41.9)	46 (29.5)	
PD	32 (38.5)	23 (44.2)	9 (29.1)	89 (57.0)	
ORR (%)	18.1	11.6	29.0	13.5	0.056
DCR (%)	61.5	55.8	70.9	43.0	0.005
Lung	*N* = 51	*N* = 35	*N* = 16	*N* = 83	
CR	0 (0.0)	0 (0.0)	0 (0.0)	9 (10.8)	
PR	9 (17.7)	4 (11.4)	5 (31.3)	12 (14.5)	
SD	12 (23.5)	5 (14.3)	7 (43.7)	48 (57.8)	
PD	30 (58.8)	26 (74.3)	4 (25.0)	14 (16.9)	
ORR (%)	17.7	11.4	31.3	25.3	0.225
DCR (%)	41.2	25.7	75.0	83.1	0.482
Lymph nodes	*N* = 30	*N* = 18	*N* = 12	*N* = 28	
CR	0 (0.0)	0 (0.0)	0 (0.0)	0 (0.0)	
PR	9 (30.0)	5 (27.8)	4 (33.3)	11 (39.3)	
SD	12 (40.0)	9 (50.0)	3 (25.0)	13 (46.4)	
PD	9 (30.0)	4 (22.2)	5 (41.7)	4 (14.3)	
ORR (%)	30.0	27.8	33.3	39.3	1.000
DCR (%)	70.0	77.8	58.3	85.7	0.097
Other sites	*N* = 40	*N* = 27	*N* = 13	*N* = 38	
CR	3 (7.5)	1 (3.7)	2 (15.4)	2 (5.3)	
PR	2 (5.0)	1 (3.7)	1 (7.7)	5 (13.2)	
SD	23 (57.5)	16 (59.3)	7 (53.9)	17 (44.7)	
PD	12 (30.0)	9 (33.3)	3 (23.0)	14 (36.8)	
ORR (%)	12.5	7.4	23.1	18.4	0.404
DCR (%)	70.0	66.7	76.9	63.2	0.323

Abbreviations: CR, complete response; DCR, disease control rate; ICI, immune checkpoint inhibitor; Nivo/Ipi, Nivolumab + Ipilimumab; Nivo, Nivolumab; ORR, objective response rate; PD, progressive diseases; PR, partial response; SD, stable disease.

* *p*‐values were calculated using Fisher's exact test due to small sample sizes.

### 3.3 Organ‐Specific Response Patterns and Survival Outcomes in the Nivo/Ipi Treatment Group

**FIGURE 3:**
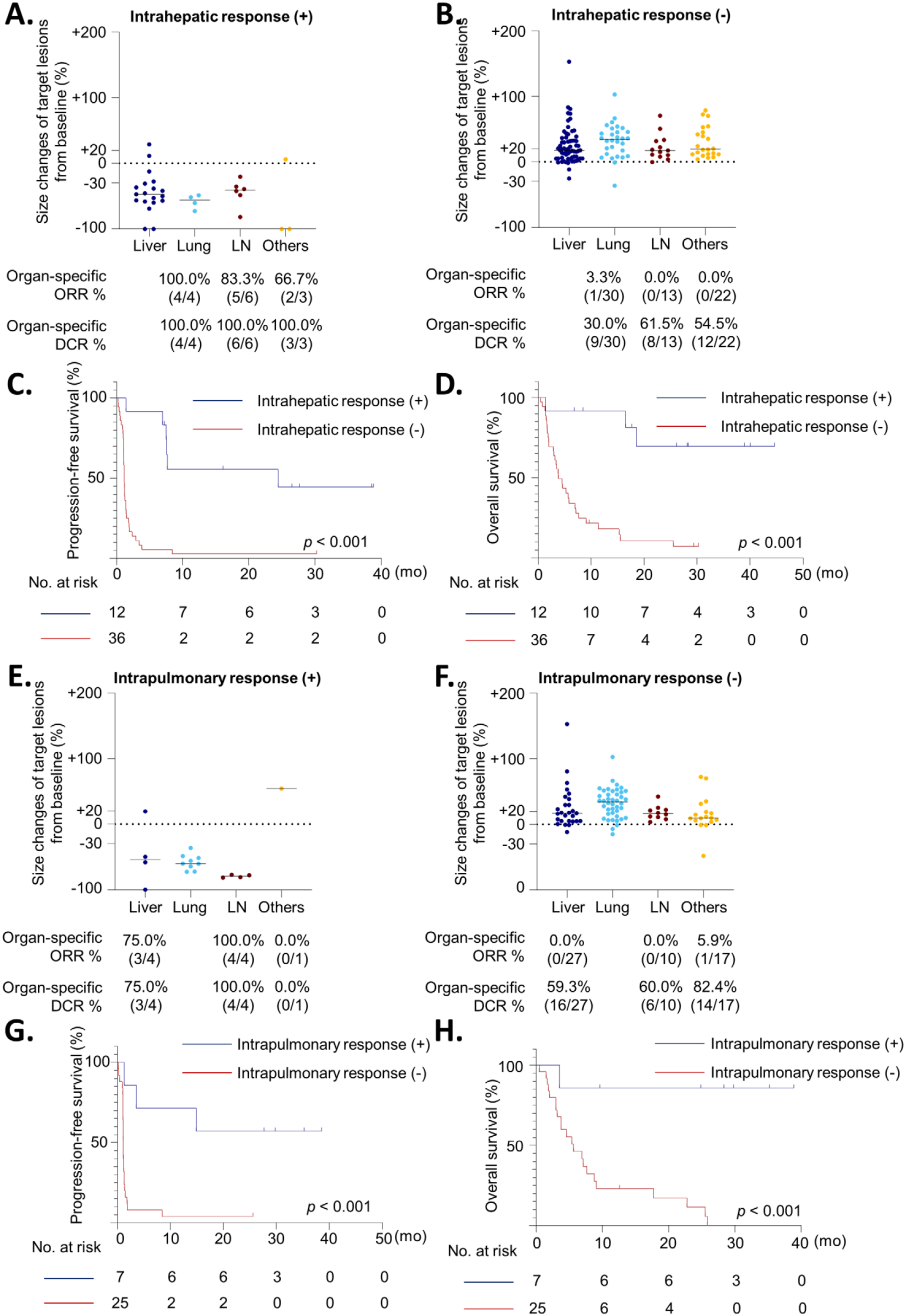
Organ‐specific response of other organs and survival outcomes of organ‐specific responders in the Nivo/Ipi treatment group. (A) Organ‐specific response of intrahepatic responders (B) Organ‐specific responders of intrahepatic non‐responders (C, D) PFS and OS based on intrahepatic response. (E) Organ‐specific response of intrapulmonary responders (F) Organ‐specific responders of intrapulmonary non‐responders (G, H) PFS and OS based on intrapulmonary response. PFS, Progression‐free survival; OS, overall survival.

In the Nivo/Ipi group, patients who responded in at least one organ were more likely to have responses in other organs, regardless of the organ type (Figure 3). Among patients with intrahepatic responses to Nivo/Ipi treatment, the OSORRs for other organs were: 100.0% (4/4) in the lungs, 83.3% (5/6) in LNs, and 66.7% (2/3) at other metastatic sites (Figure 3A). In contrast, in patients without intrahepatic responses to Nivo/Ipi, no response was observed in any organ other than pulmonary lesions (Figure 3B). In patients with intrapulmonary responses to Nivo/Ipi, the OSORRs for other organs were: 75.0% (3/4) in the liver, 100.0% (4/4) in LNs, and 0.0% (0/1) at other metastatic sites (Figure 3E). Conversely, in patients without intrapulmonary responses, there were no responses in any other organ except for one lesion at the other metastatic sites (Figure 3F). Of the 69 patients, only two patients showed variable responses in different organs.

Furthermore, responders in the liver or lung experienced improved survival outcomes, including longer progression‐free survival (PFS) and OS, compared with non‐responders. The median PFS was 24.4 months for patients with intrahepatic responses and 1.2 months for those without (*p* < 0.001) (Figure 3C). The median OS was not reached in intrahepatic responders, whereas intrahepatic non‐responders had a median OS of 3.8 months (*p* < 0.001) (Figure 3D). Similarly, the median PFS was not reached in intrapulmonary responders, while the median PFS was 1.2 months for intrapulmonary non‐responders (*p* < 0.001) (Figure 3G). The median OS for intrapulmonary responders was not reached, whereas non‐responders had a median OS of 5.7 months (*p* < 0.001) (Figure 3H).

## 4 Discussion

Here, we evaluated the overall ORRs and OSORRs in patients with advanced HCC by analyzing 204 individual lesions treated with Nivo/Ipi and 305 lesions treated with Nivo monotherapy across five cancer centers in Korea. To date, this is the first study to compare OSORRs between Nivo/Ipi and Nivo monotherapy in patients with advanced HCC.

Patients treated with Nivo/Ipi without prior ICI therapy demonstrated superior OSORRs, particularly in the liver, compared with those who received Nivo/Ipi with prior ICI exposure or Nivo monotherapy. Previous studies showed that the liver, an immune‐tolerant organ, often exhibits suboptimal responses to ICI treatments [12]. However, in this study, patients treated with Nivo/Ipi without prior ICI exposure demonstrated favorable intrahepatic responses, comparable to extrahepatic responses, suggesting the potential of ipilimumab to overcome liver‐specific immune tolerance.

Prolonged or repeated exposure to ICIs may lead to adaptive resistance in the tumor microenvironment. This resistance can manifest as the upregulation of alternative immune checkpoints, such as TIM‐3 or LAG‐3, or an increase in immunosuppressive cell populations, including Tregs and myeloid‐derived suppressor cells. Additionally, prior ICI exposure may lead to T‐cell exhaustion, characterized by impaired proliferation, reduced cytokine production, and diminished cytotoxicity, which could limit the efficacy of subsequent ICI therapies [18, 19].

Prior exposure to ICIs appears to have a detrimental effect on both the overall treatment efficacy and OSORRs of Nivo/Ipi‐treated patients. Patients with prior ICI exposure had markedly lower OSORRs across all assessed organs than those without prior ICI exposure. This effect was particularly evident in intrahepatic lesions, where the OSORR was significantly reduced in patients with prior ICI exposure.

Organ‐specific responses in at least one organ were strongly associated with improved survival outcomes in patients treated with Nivo/Ipi, as demonstrated by the significantly longer PFS and OS among responders than among non‐responders. Of the 69 patients, only two patients showed variable responses in different organs. Intrahepatic responders experienced particularly notable survival benefits, with a median PFS of 24.4 months and a median OS not reached, compared to 1.2 and 3.8 months, respectively, in non‐responders. Similarly, intrapulmonary responders showed superior survival outcomes, with a median PFS and OS not reached, whereas non‐responders had a median PFS of 1.2 months and a median OS of 5.7 months. These findings indicate that the response in a single organ may be significantly associated with substantial survival benefits in patients treated with Nivo/Ipi.

Liver‐specific immune tolerance poses a significant challenge in achieving effective immunotherapeutic responses in HCC. Preclinical studies demonstrated that the depletion of Tregs or intrahepatic macrophages can activate peripheral CD8+ T‐cell immunity and induce intrahepatic tumor regression, suggesting the potential of liver‐directed immunotherapy [10, 11]. Ipilimumab, an anti‐CTLA‐4 inhibitor, has emerged as a promising therapeutic option when combined with anti‐PD‐1/PD‐L1 agents in advanced HCC, as evidenced by recent phase III trials such as CheckMate 9DW and HIMALAYA [16]. Mechanistically, anti‐CTLA‐4 antibodies act through three primary pathways: enhancing effector T‐cell activity by blocking the CD80/86‐CTLA‐4 interaction, inducing antibody‐dependent cellular cytotoxicity and phagocytosis to deplete Tregs, and altering Treg metabolism from oxidative phosphorylation to glycolysis via CD28 and CD80/86 co‐stimulatory signaling, thereby reducing Treg‐mediated immunosuppression [20]. Collectively, these mechanisms modify the immunosuppressive tumor microenvironment, enabling Nivo/Ipi combination therapy to overcome liver‐specific immune tolerance and achieve an enhanced response, particularly in intrahepatic lesions. This highlights the potential of anti‐CTLA‐4 agents to reprogram the immune environment of the liver and offers a viable strategy for improving immunotherapeutic outcomes in HCC.

This study has several limitations. First, its retrospective nature and the relatively small sample size limit the robustness of our conclusions and the generalizability of the findings. Second, the patients were of East Asian ethnicity, and the study was conducted in HBV‐endemic regions. Third, new lesions, as defined by the RECIST guidelines, were not considered in the organ‐based response evaluation due to the organ‐specific nature of OSORR assessment. Finally, we did not investigate the immunological mechanism of action of Nivo/Ipi in the hepatic microenvironment. Despite these limitations, our study offers valuable insights into the organ‐specific responses to Nivo/Ipi in patients with advanced HCC.

To the best of our knowledge, this study is the first to demonstrate that Nivo/Ipi combination therapy can achieve intrahepatic responses in advanced HCC comparable to those observed with atezolizumab plus bevacizumab, a well‐established first‐line regimen. These findings highlight the potential of Nivo/Ipi as a viable first‐line treatment option and underscore the importance of assessing organ‐specific responses across different therapeutic regimens, as such evaluations may offer valuable insights for optimizing patient outcomes and refining treatment strategies.

In conclusion, this study demonstrated that Nivo/Ipi combination therapy achieved superior intrahepatic responses compared with Nivo monotherapy in patients with advanced HCC without prior ICI exposure, highlighting its potential to overcome liver‐specific immune tolerance.

## Author Contributions

**Jung Sun Kim:** writing – original draft, writing – review and editing, conceptualization, methodology, software, data curation, supervision, formal analysis, validation, investigation, visualization, project administration, resources. **Youngun Kim:** conceptualization, investigation, writing – original draft, writing – review and editing, visualization, validation, methodology, software, formal analysis, project administration, supervision, data curation. **Beodeul Kang:** resources, data curation, writing – review and editing. **Ilhwan Kim:** writing – review and editing, resources, data curation. **Hyeyeong Kim:** resources, data curation, writing – review and editing. **Won Suk Lee:** writing – review and editing, resources, data curation. **Jung Yong Hong:** writing – review and editing, resources, data curation. **Ho Yeong Lim:** data curation, resources, writing – review and editing. **Han Sang Kim:** writing – review and editing, resources, data curation. **Chang Gon Kim:** data curation, resources, writing – review and editing. **Sanghoon Jung:** writing – review and editing, resources, data curation. **Chansik An:** conceptualization, investigation, writing – original draft, writing – review and editing, visualization, validation, methodology, software, formal analysis, project administration, resources, supervision, data curation. **Chan Kim:** data curation, resources, writing – review and editing, conceptualization, investigation, funding acquisition, writing – original draft, visualization, validation, methodology, writing – review and editing, project administration, formal analysis, software, data curation, supervision, resources. **Hong Jae Chon:** data curation, supervision, resources, project administration, formal analysis, software, methodology, validation, visualization, writing – review and editing, writing – original draft, funding acquisition, investigation, conceptualization.

## Ethics Statement

This study protocol was reviewed and approved by the institutional review board of each participating center (CHA Bundang Medical Center, approval number 2019‐10‐009, 2024‐07‐063; Yonsei Cancer Center, 4‐2019‐0882; Samsung Medical Center, 2019‐10‐063; Haeundae Paik Hospital, 2019‐10‐025‐001; and Ulsan University Hospital, 2019‐11‐031‐002) and was performed in accordance with the ethical standards of the institutional research committee and the recent Declaration of Helsinki.

## Consent

The need for informed consent in this study was waived, as Korean regulations do not require consent for retrospective analyses.

## Conflicts of Interest

Hong Jae Chon consults for Eisai, Roche, Bayer, ONO, MSD, BMS, Celgene, Sanofi, Servier, AstraZeneca, SillaJen, Menarini, and GreenCross Cell, and has research grants from Roche, Dong‐A ST, and Boryung Pharmaceuticals. Chan Kim consults for Roche, ONO, MSD, BMS, Oncocross, and Virocure, and receives research funding from Boryung Pharmaceuticals, Oncocross, SillaJen, and Virocure.

## Supporting information


**Figure S1.** Organ‐specific response in the Nivo/Ipi group and the Nivo group. (A) Liver‐specific response (B) Lung‐specific response (C) Lymph node‐specific response (D) Other metastatic lesion (bone and brain)‐specific response.

## Data Availability

These data are not publicly available due to ethical reasons. Further inquiries can be directed to the corresponding authors.
