# Supplementary figures and images for: Organ‐Specific Responses to Nivolumab Plus Ipilimumab in Advanced Hepatocellular Carcinoma: A Multicenter, Retrospective Study
Source: Cancer Med. 2025 Jun 9;14(11):e70997. doi: 10.1002/cam4.70997 (PMC12146901; doi:10.1002/cam4.70997)

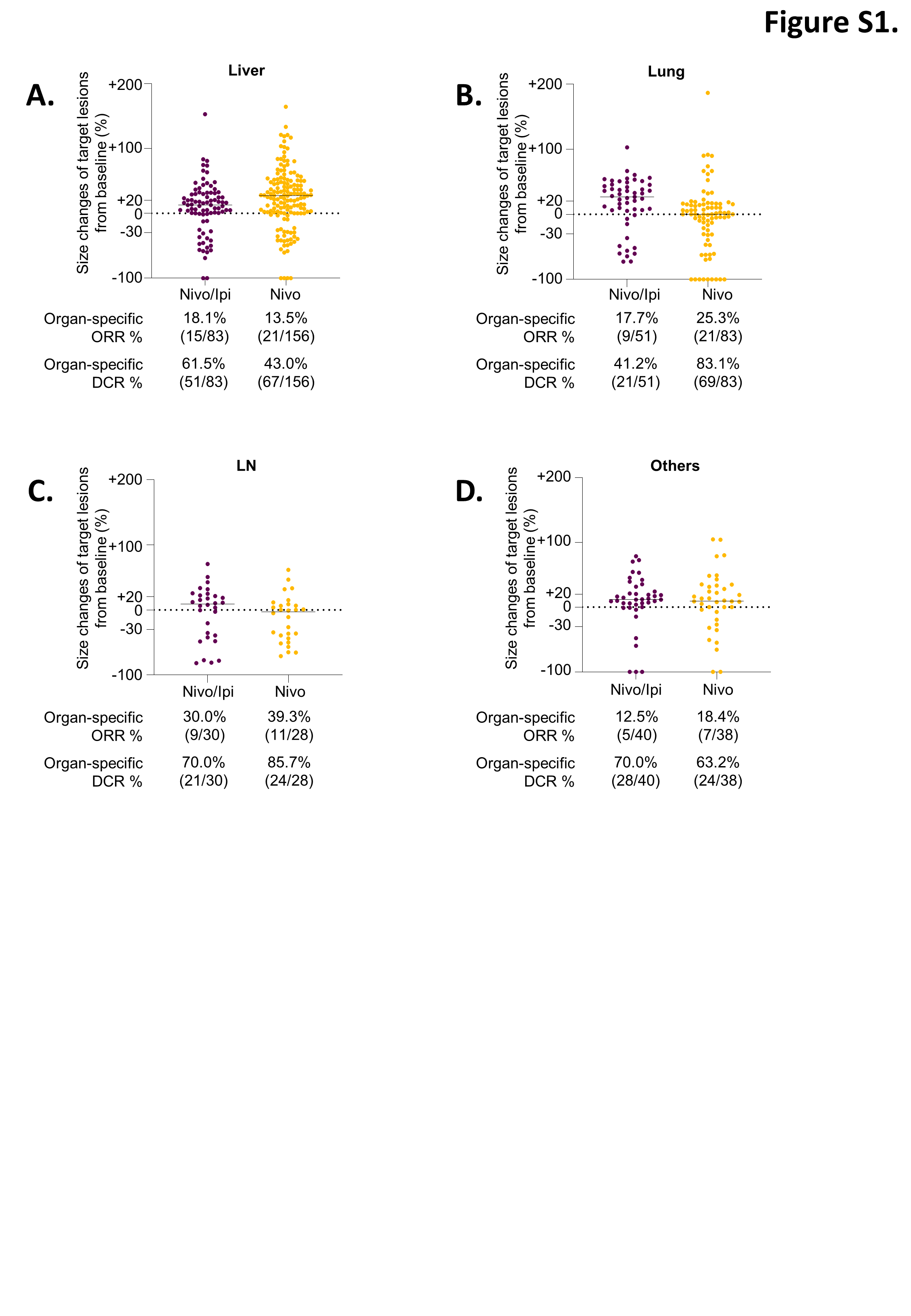

Supplement: Supplementary file 1 — Figure S1. Organ‐specific response in the Nivo/Ipi group and the Nivo group. (A) Liver‐specific response (B) Lung‐specific response (C) Lymph node‐specific response (D) Other metastatic lesion (bone and brain)‐specific response. [file CAM4-14-e70997-s001.tif]
